# Prevalence of Chlamydia in Young Adulthood and Association with Life Course Socioeconomic Position: Birth Cohort Study

**DOI:** 10.1371/journal.pone.0104943

**Published:** 2014-08-25

**Authors:** Joanna Crichton, Matthew Hickman, Rona Campbell, Jon Heron, Paddy Horner, John Macleod

**Affiliations:** School of Social and Community Medicine, University of Bristol, Bristol, United Kingdom; University of California Merced, United States of America

## Abstract

**Background:**

Few estimates are available of chlamydia prevalence in the general population. Existing studies have limited scope to explore potential selection bias or associations with socioeconomic position.

**Methods:**

We examined the prevalence of *Chlamydia trachomatis* infection and associations with life-course socioeconomic position in the Avon Longitudinal Study of Parents and Children in England. Chlamydia infection was measured through nucleic acid amplification test of urine specimens.

**Results:**

4864 (51%) of those invited attended the clinic (mean age 17.8; SD 0.37 years). (60%) provided a urine specimen. Prevalence was 1.0% (95% CI 0.6 to 1.6) among participants reporting sexual activity. Risk of infection was strongly associated with life course social disadvantage and with recent sexual behaviour. After adjustment for other measures of disadvantage and for sexual behaviour the strongest risk factors for infection were lower maternal educational attainment (OR 9.1 (1.1, 76.7)) and lower participant educational attainment at age 11 (OR 5.0 (1.5, 16.5)). Both clinic attendance and agreement to test were lower amongst the disadvantaged. Adjustment for selective participation based on detailed information on non-participants approximately doubled prevalence estimates. Prevalence was higher in sexually active women (1.4% (0.7 to 2.4) than men (0.5% (0.1 to 1.3)).

**Conclusions:**

Chlamydia prevalence in this general population sample was low even after adjustment for selective participation in testing. These estimates of prevalence and patterns of association with socioeconomic position may both reflect recent screening efforts. Prevalence was higher amongst the disadvantaged who were also less likely to engage in testing. Our results reveal the importance of monitoring and addressing inequalities in screening programme participation and outcomes.

## Introduction

Chlamydia control policy faces challenges due to gaps in the evidence base on the natural history of *Chlamydia trachomatis* (chlamydia) and on the epidemiology of infections [1]–[3]. Since 2003, a National Chlamydia Screening Programme (NCSP) has operated in England with the aim of providing regular screening for genital chlamydia infection to all sexually active 15 to 24 year olds. Estimates of population prevalence of chlamydia in England before and after the introduction of screening are limited. Two large population-based studies were undertaken between 1999 and 2002. The National Study of Sexual Attitudes and Lifestyle (Natsal-2) [4], estimated prevalence among 18–24 year olds to be 3.0% (1.7 to 5.0) in women and 2.7% (95% CI 1.2 to 5.8) in men. In contrast, the Chlamydia Screening Studies (CLaSS) estimated prevalence amongst 16 to 19 year olds to be 6.0% (4.6 to 8.4) in women and 3.5% (2.3 to 5.2) in men [5]. A third round of Natsal, carried out in 2010–2012, looked in more detail within the 16–24 year age group and found considerable variation by age, with prevalence peaking at 18–19 years in women (4.7% (2.4, 8.6)), but remaining low in men aged under 20 (0.3% (0.1, 1.3)) before peaking at age 20–24 years [6]. In all these surveys, less than 50% of eligible individuals were tested. If participation in testing is associated with risk of infection, prevalence estimates will be biased. Depending on patterns of selective participation such bias may lead to under or over estimates of prevalence. None of the above studies had sufficient information on non-responders to allow detailed investigation of the influence of such bias.

Existing evidence on the association between socioeconomic position and risk of chlamydia is mixed, with most studies finding only weak evidence of an association between increased risk and social disadvantage [7], [8]. The most recent survey in the UK (Natsal-3), undertaken several years after the introduction of screening, found stronger evidence of such health inequality with risk of infection amongst residents of the most disadvantaged areas being around double that of participants from the least disadvantaged areas in both men and women aged 16–44 years. This study did not examine whether this relationship was also true in the 16–24 age group targeted by the NCSP [6]. ClaSS found some evidence that women at higher risk of infection were harder to engage in testing. Any effective health intervention may exacerbate health inequality if uptake of the intervention is higher amongst individuals at lower risk [9], [10].

We estimated prevalence of chlamydia infection amongst 16–19 year old participants in the Avon Longitudinal Study of Parents and Children (ALSPAC). We investigated the association between risk of infection and both life course socioeconomic position and recent sexual behaviour. Using information on individuals eligible to be tested for chlamydia but not tested we also investigated the influence of bias arising through selective participation on prevalence estimates.

## Methods

The Avon Longitudinal Study of Parents and Children (ALSPAC) is an ongoing, population-based, prospective cohort study examining health and socioeconomic data on children and their parents from the child's gestation to early adulthood. ALSPAC sought to recruit all pregnant women resident in the Bristol area of the UK during 1991–92. This area (1991 total population ∼0.9 million) included the city of Bristol, which had a population of approximately 0.5 million in 1991, and surrounding urban and rural areas, including towns, villages and farming communities [11]. The study recruited 14 541 women, who gave birth to 14 062 children, of whom 13 988 were alive at one year of age. All participants gave informed consent. Ethical approval for the study was granted by the ALSPAC Law and Ethics Committee. Detailed descriptions of methods and participants have already been published [11] and a fully searchable dictionary of available data is available [12].

**Figure 1:**
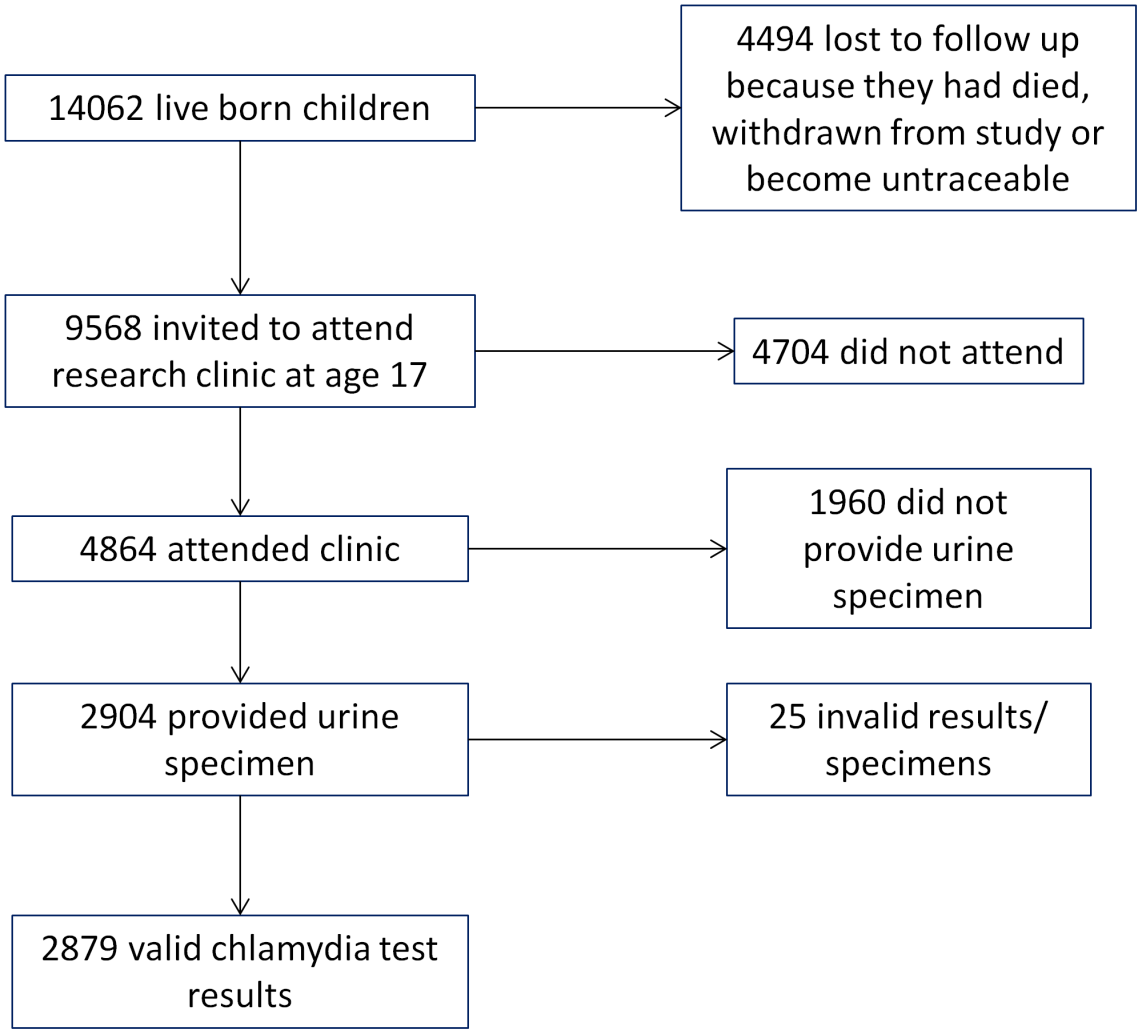
Flowchart of inclusion and attrition from ALSPAC chlamydia prevalence study.

Between December 2008 and June 2011, 9568 eligible (see figure 1) participants were invited to attend a research clinic at approximately 17 years of age. Initial invitations were by post and numerous attempts were made to reach non-responders, including reminder letters, telephone calls and outreach in schools and colleges. Transport and accommodation costs were reimbursed and participants were offered shopping vouchers as compensation for taking part.

Clinic participants were offered tests for chlamydia and gonorrhoea in partnership with the NCSP. They were asked to report whether they were sexually active with the question ‘Have you ever had sexual intercourse with either a female (woman/girl) or a male (man/boy))?’ (Yes/No). Those answering in the affirmative were asked to report their number of partners in the previous year, and this variable was used as a measure of recent sexual behaviour in this study. Participants were invited to provide a first catch urine specimen irrespective of their reported sexual activity and to provide contact details. They were informed that they would be notified of their test result and that if it was positive, treatment and partner notification would be provided through the National Chlamydia Screening Programme (NCSP). Samples were tested at the local Health Protection Agency laboratory (now Public Health England) using the Gen-Probe Aptima Combo 2 (AC2) Assay (San Diego, California, USA), which detects both *C. trachomatis* and *Neisseria gonorrhoeae*. All chlamydia-positives were confirmed using the Gen-Probe Aptima CT assay. The local screening office of the NCSP were informed of all results, which were managed according to their normal protocol.

Mothers of the young people provided measures of household socioeconomic position (parent's highest occupational class and maternal education) and multiple measures of family adversity during pregnancy. Disposable household income was calculated from information provided by mothers when the child was aged 33 to 47 months [13]. Data on the child's free school meal eligibility, Special Educational Needs Status, and educational attainment during national tests at age 10–11 years and 15–16 were obtained from the National Pupil Database. Free school meals and Special Educational Needs status are allocated by schools to identify students with socio-economic disadvantage and learning difficulties or disability, respectively. Performance was assessed according to National Curriculum target achievement levels and converted into binary variables. The young person's postcode at the time of the age 17 clinic was linked to the Index of Multiple Deprivation (IMD) score of their neighbourhood, which was mapped to national deciles and converted into a binary measure cut at the lowest 25%.

The number of young people eligible for follow up at age 17–19 was 9568, of whom 4864 (51% of those eligible) attended the clinic. Urine specimens for testing for chlamydia infection were provided by 2904 (60%) of clinic participants (figure 1). Measures of socioeconomic position and education were available for between 84 and 98% of those who tested (table 1).

**Table 1:** Completeness of data among young people who tested for chlamydia at the research clinic and those who were invited to the clinic.

		Chlamydia testers (n = 2879)		Eligible to attend (n = 9568)	
Timing	Measure	Values observed	%	Values observed	%
	**Outcome**				
Late teens	Chlamydia test result	2879	100	2879	30
	**Explanatory variables**				
Gestation/	Gender	2879	100	9568	100
infancy	Mother's education	2788	97	8839	92
	Parent's highest social class	2673	93	8293	87
	Household Disposable income	2548	89	7609	80
Early teens	Free school meals eligibility	2491	87	8247	86
	Educational attainment at age 10-11	2535	88	8232	86
Mid teens	Educational attainment at Key Stage 4 (GCSE/equivalent)	2502	87	8184	86
Late teens	Neighbourhood disadvantage (IMD) at Age 17 clinic	2820	98	2846	30
	Number of sexual partners in the past year	2419	84	3803	40

We calculated prevalence of chlamydia infection for all participants with a chlamydia test result, by gender and among those reporting ever having sex. We investigated unadjusted associations between the outcome, chlamydia infection, and a series of measures of life course socioeconomic position and sexual behaviour using logistic regression. In multivariable analysis, we examined the strength of associations between individual socioeconomic position measures and risk of infection whilst adjusting for age and other explanatory variables, considering potential confounding variables within a hierarchical conceptual framework [14]. We further adjusted for self-reported number of sexual partners in the past year, to examine the extent that any observed associations of chlamydia infection with socioeconomic position or gender may be explained by differences in sexual behaviour.

We investigated the possible influence of selective participation on our estimates of chlamydia prevalence using both multiple imputation and inverse probability weighting [15], [16]. In the former, the variables in the analysis model and 19 other measures of socioeconomic position and family adversity were imputed for all 9568 individuals eligible to attend the clinic. We generated 100 imputed datasets, and combined estimates using Rubin's rules [16]. In the latter, estimates of prevalence and associations were weighted to account for probabilities of non-response to the interview and chlamydia test [15]. These weights were derived from logistic regression models using variables associated with non-response, including gender, family adversity, socioeconomic position and birthweight. In a third sensitivity test, we combined the two methods using the approach suggested by Seaman et al [17] for dealing with attrition and missing data in cohort studies (IPW/MI). We imputed missing values among the 4864 young people who attended the research clinic, then weighted the combined estimates to account for those who did not attend the clinic. Analyses were carried out using STATA 12 [18].

## Results

A total of 2904 individuals provided samples for testing for chlamydia, from which 2879 valid results were obtained. Ninety seven percent (2792) of those providing valid samples were aged 17–18 years (range 16.3–19.8, mean 17.8, standard deviation 0.37). Fifty four percent (1541) were women. Fifty five percent (1595) of individuals providing valid samples reported that they had ever had sex An additional 1384 (70%) of those who attended the clinic but did not provide a sample answered the question on sexual experience. Thirty one percent of these non-testers reported that they were sexually active. 54% of female testers and 44% of male testers reported not using a condom at last sex.

Refusing to provide a urine sample at the clinic was associated with being female (odds ratio 0.8 (95% confidence interval 0.7, 0.9), p<0.001), lower educational attainment at age 16 (OR 0.6 (0.5, 0.7) p<0.001) and measures of family socioeconomic disadvantage (results not shown). Socio-economic and educational disadvantage was higher among those at the age 17 clinic who declined to provide a sample and highest among those who missed the clinic entirely (table 2).

**Table 2:** Measures of socioeconomic and educational disadvantage, comparing i) participants who did and did not attend the research clinic and ii) clinic attendees who did and did not take the chlamydia test.

	Attended clinic			Took chlamydia test at clinic		
	No (N = 4704)	Yes (N = 4864)	χ^2^, p	No (N = 1985)	Yes (N = 2879)	χ^2^, p
Measure of disadvantage	n(%^a^)	n(%^a^)		n(%^a^)	n(%^a^)	
Parent with no qualification	663 (14)	437 (9)	61.4, <0.001	208 (10)	229 (8)	9.2, 0.002
Parent occupation manual/skilled non-manual	1825 (48)	1635 (36)	111.0, <0.001	708 (39)	927 (35)	9.1, 0.003
Free School Meals Eligibility	704 (18)	305 (7)	204.6, <0.001	135 (8)	170 (7)	1.3, 0.250
Disposable household income lowest 20%	779 (23)	597 (14)	110.6, <0.001	265 (15)	332 (13)	4.8, 0.029
Special educational needs b	178 (4)	99 (2)	29.5, <0.001	51 (3)	48 (2)	5.0, 0.026
Did not meet educational attainment targets at age 10–11	1282 (33)	728 (17)	227.2, <0.001	368 (21)	360 (14)	31.8, <0.001

a Percentages using all observed values for each variable.

b Special Educational Needs (SEN) status at one or more timepoint (age 7, 12–13 and/or 13–14 years).

All samples tested negative for gonorrhoea, 20 test results were positive for chlamydia. Prevalence was 0.7% (0.4 to 1.1) among all testers and 1.0% (0.6 to 1.6) among those who reported ever having sex (table 3). Prevalence was higher in women than men (1.4% (0.7 to 2.4) vs. 0.5% (0.1 to 1.3) of sexually active women and men).

**Table 3:** Estimates of prevalence of chlamydia Infection i) for all participants and ii) those reporting sexual activity, by gender, with and without sensitivity test for bias due to missing data.

	Complete case			Multiply-imputed (100 imputations)^a^
Denominator:	N	Positive	% positive (95% CI)	% positive (95% CI)
All young people				
All	2,879	20	0.7 (0.4, 1.1)	2.0 (1.0, 2.9)
Women	1541	15	1.0 (0.6, 1.6)	2.4 (1.1, 3.6)
Men	1338	5	0.4 (0.1, 0.9)	1.6 (0.5, 2.7)
Reported sexual activity^b^				
All	1591	16	1.0 (0.6, 1.6)	
Women	935	13	1.4 (0.7, 2.4)	
Men	656	3	0.5 (0.1, 1.3)	

Notes: ^a^Data were multiply imputed for the 9568 participants who attended the study clinic.

b The number of cases of chlamydia among participants reporting sexual activity is 16, rather than 20, because four participants who tested positive for chlamydia did not respond to the question on sexual activity.

In unadjusted analyses, disadvantage in most measures of socioeconomic position and education was associated with chlamydia infection (table 4). The strongest and most substantial associations were seen with maternal education (odds ratio 5.5 95% confidence interval 1.6 to 18.8; O Level or vocational qualifications only compared to A level or higher); parental occupational class (OR 3.8, CI 1.4 to 10.2; occupational class III-V compared to I-II); parental income (OR 4.8, CI 1.8 to 12.6; lowest compared to other quintiles) and participants' educational achievement (OR 6.2, CI 2.3–16.5; failed to achieve target level in tests at age 10–11 years). Fewer GCSE qualifications (national examinations entered by pupils in the UK at 16 years of age) also predicted chlamydia infection (results not shown). Risk of infection was strongly associated with increasing reported number of sexual partners in the past year.

**Table 4:** Unadjusted associations between chlamydia infection and gender, socioeconomic position, education and number of sexual partners.

		N a	Positive	Positivity (%) (95% CI)	Unadjusted OR (95% CI)	p
Sex	Male	1338	5	0.6 (0.2, 1.7)	1	0.047
	Female	1541	15	1.0 (0.6, 1.7)	2.6 (1.0, 7.2)	
Maternal education	A-Level/Degree	1,365	3	0.2 (0.1, 0.6)	1	0.001
	O-Level/CSE/vocational	1423	17	1.2 (0.7, 1.9)	5.5 (1.6, 18.8)	
Occupational Class	i/ii	1,746	6	0.3 (0.1, 0.7)	1	0.006
	iii (nm+m)/iv/v	927	12	1.0 (0.1, 3.5)	3.8 (1.4, 10.2)	
Disposable Income	Highest 80%	2,206	10	0.4 (0.2, 0.8)	1	0.004
(quintiles)	Lowest 20%	325	7	2.1 (0.9, 4.3)	4.8 (1.8, 12.6)	
Free School Meals	No	2,321	14	0.6 (0.3, 1.0)	1	0.132
	Yes	170	3	1.8 (0.4, 5.1)	3.0 (0.8, 10.4)	
Achieved target National Curriculum Level in all three core subjects at KS2	Yes	2,175	8	0.4 (0.2, 0.7)	1	
	No	360	8	2.2 (1.0, 4.3)	6.2 (2.3, 16.5)	<0.001
Index of Multiple Deprivation	Less Deprived 75%	2121	10	0.5 (0.2, 0.9)	1	0.033
	Highest 25% of deprivation scores	699	9	1.3 (0.6, 2.4)	2.8 (1.1, 6.8)	
Number of sexual partners in the past year	0 or 1	1,827	7	0.4 (0.2, 0.8)	1	0.014
	2	285	3	1.0 (0.2, 3.0)	2.8 (0.7, 10.7)	
	3 or more	291	6	2.0 (0.7, 4.3)	5.4 (1.8, 16.1)	

a Unadjusted analyses using all available data. 2879 participants have valid results from chlamydia tests at the clinic. Counts for some covariables add up to less than 2879 due to missing values of exposures.

After adjusting for potential confounding by age and other explanatory variables, the associations between chlamydia infection and young person's educational attainment and income were attenuated slightly but remained strong (OR 5.0 (1.6, 15.5) and 3.1 (1.1, 9.1), respectively) (table 5). The odds ratio for greater risk of chlamydia infection in those with lower maternal education (adjusted OR 4.1 (0.8 to 20.6)) remained relatively large but with wide confidence intervals consistent with no relationship. Further adjusting for reported number of sexual partners strengthened the association with maternal education (OR 9.1 (1.1, 76.7)) and did not affect the magnitude of association with young person's educational attainment, while associations with occupation and class were weakened.

**Table 5:** Adjusted associations between chlamydia infection and gender, socioeconomic position, education and number of sexual partners, with complete case analysis and using three sensitivity tests examining missing data.

	Complete case: participants with chlamydia test result (N = 2879^a^) OR (95% CI) p value			Multiply Imputed in participants invited to clinic (N = 9568) OR (95% CI) p value		
	Unadjusted	Adjusted for potential confounding^b^	Adjusted for confounding & sexual behaviour^c^	Unadjusted	Adjusted for potential confounding^b^	Adjusted for confounding & sexual behaviour^c^
**Mother's educational qualifications**						
A Level/Degree Vocational/O Level or less	1	1	1	1	1	1
	5.5 (1.6, 18.8)	4.1 (0.8, 20.6)	9.1 (1.1, 76.7)	3.6 (1.6, 8.4)	2.3 (0.9, 5.7)	2.2 (0.9, 5.6)
	p = 0.001	p = 0.083	p = 0.042	p = 0.003	p = 0.082	p = 0.102
**Parental occupational class**						
i/ii	1	1	1	1	1	1
iii (nm& m)/iv	3.8 (1.4, 10.2)	1.9 (0.6, 6.0)	1.3 (0.4, 4.2)	2.8 (1.4, 5.6)	1.7 (0.8, 3.6)	1.6 (0.7, 3.5)
	p = 0.008	p = 0.293	p = 0.663	p = 0.005	p = 0.210	p = 0.258
**Family disposable income**						
Not in lowest quintile	1	1	1	1	1	1
Lowest quintile	4.8 (1.8, 12.6)	3.1 (1.1, 9.1)	1.8 (0.5, 6.1)	3.4 (1.7, 7.1)	2.5 (1.1, 5.4)	2.4 (1.1, 5.3)
	p = 0.002	p = 0.036	p = 0.337	p = 0.001	p = 0.022	p = 0.028
**Young person**'**s educational attainment**						
Achieved target at age 10/11	1	1	1	1	1	1
Did not achieve target	6.2 (2.3, 16.5)	5.0 (1.6, 15.5)	5.0 (1.5, 16.5)	4.0 (1.7, 9.6)	2.9 (1.2, 7.2)	3.0 (1.2, 7.4)
	p<0.001	P = 0.005	p = 0.008	p = 0.002	p = 0.022	p = 0.019
**Gender**						
Male	1	1	1	1	1	1
Female	2.6 (0.9, 7.2)	2.6 (0.9, 7.2)	3.5 (1.0, 12.4)	1.5 (0.7, 3.4)	1.5 (0.7, 3.4)	1.4 (0.6, 3.2)
	p = 0.063	p = 0.063	p = 0.051	p = 0.287	p = 0.307	p = 0.363
**Sexual partners in past year**						
0-1 sexual partners	1			1		
2+ sexual partners	4.1 (1.5, 11.0)			2.6 (1.2, 5.7)		
	p = 0.005			p = 0.020		

a N varies between 1809 and 2879 due to missing values of measures of socioeconomic position and number of sexual partners.

b Model 1: Adjusted for age and other measures of socio-economic position that were considered to be potential confounders, identified using a hierarchical conceptual framework.

c Model 2: Adjusted for variables in Model 1 and reported number of sexual partners.

Multiple imputation of missing values led to substantial increases in prevalence estimates, to 2.0 (1.0, 2.9) in all participants, 2.4 (1.1, 3.6) in women and 1.6 (0.5, 2.7) in men (table 3). Prevalence estimates obtained from further missingness analyses involving inverse probability weighting were similar to those in multiply-imputed data (not shown). In multiply-imputed data, the magnitude of estimates of association of both socioeconomic position and sexual behaviour with risk of infection was diminished slightly in both the weighted and imputed datasets. However the pattern of association was unchanged and associations generally remained strong (table 5). Estimates of both prevalence and relative risk were imprecise reflecting the small number of cases.

## Discussion

In this large population-based sample of young adults within the target age range of the National Chlamydia Screening Programme, chlamydia prevalence was 2.4% (1.1, 3.6) in women and 1.6% (0.5, 2.7) in men after adjustment for bias introduced by selective participation. Such bias was considerable as prevalence estimates were approximately doubled by adjustment; this adjustment was not possible in other surveys of population chlamydia prevalence where only very limited information on non-participants was available. We confirmed associations between risk of infection and number of sexual partners in the past year. We also found strong evidence of social inequalities in chlamydia. After adjusting for social differences in sexual behaviour (measured by number of sexual partners), participants whose mothers had the lowest level of educational attainment were almost ten times more likely to test positive than participants whose mothers had the highest level of educational achievement. Participants who themselves had the lowest level of educational attainment at age 11 were five times more likely to test positive than participants with the highest level. Acceptance of an invitation for both the general ALSPAC clinical assessment and the specific invitation to participate in chlamydia screening were both lower amongst the disadvantaged.

Strengths of this study included its large population sample, the richness of available measures of life course socioeconomic position and the extent of prospectively collected information on possible risk factors for both individuals tested and those eligible but not tested, including information obtained from national administrative databases and thus relatively complete. This allowed detailed investigation both of influences on infection risk and of possible influences of selective participation on prevalence estimates. Slightly more than half of those eligible attended for assessment and of these slightly less than two thirds provided a urine sample for testing. This notwithstanding, we tested almost 3,000 16–19 year olds – considerably more than other recent population-based surveys, and with similar response rates (Table 6) [4]–[6]. Unfortunately it was not possible to record reasons for refusal to take a chlamydia test, due to wider study considerations. The small number of positive cases we identified meant that prevalence estimates and most observed associations with possible risk factors were imprecise. Despite the small number of cases, associations with socioeconomic position and education measures were strong, and generally remained so after imputation of missing data. This is in keeping with the generally accepted view in observational epidemiology that whilst missing data due to selective participation may often bias estimates of prevalence it is unlikely to bias estimates of effects on exposure-outcome associations except in unusual situations [19].

**Table 6:** Recent UK population based studies of prevalence of chlamydia in the general population.

Study	Age range	% of eligible sample with chlamydia result	Number tested	Number positive	Prevalence in women (%)^a^	Prevalence in men (%)^a^
Natsal-2 (1999–2001)	18–24	46% (all participants aged 16–44)	186	6	3.0 (1.7, 5.0)	2.7 (1.2, 5.8)
ClaSS (2001–2)	16–19	30%	1577	77	6.0 (4.6, 8.4)	3.5 (2.3, 5.2)
Natsal-3 (2010–2012)	16–19	38% (all participants aged 16–44)	738	Not reported	3.8 (2.2, 6.3)	0.3 (0.1, 1.3)
ALSPAC (2008–2011)	16–19	31%	2879	20	Complete case:	
					1.0 (0.6, 1.6)	0.4 (0.1, 0.9)
					Adjusted for missing data:	
					2.4 (1.1, 3.6)	1.6 (0.5, 2.7)

a Natsal and ClaSS estimates weighted by sampling probabilities, Natsal 2010 additionally weighted for test refusal. ALSPAC adjusted prevalence estimates based on multiply-imputed data.

Prevalence of genital chlamydia in our study was lower than that found in population-based surveys from a decade ago [4], [5], and from pooled prevalence estimates from an evidence synthesis study [20]. Estimates from a more recent survey conducted over a similar time period were more similar to our own [6]. There are several possible reasons for these apparent discrepancies. True prevalence may vary between different study populations and prevalence may also have changed over time, including as a result of screening efforts. Other, indirect evidence is consistent with this latter explanation [21], [22]. Other comparable surveys had similar response rates to our own (table 6) and it is thus similarly likely that selective participation led to bias in their prevalence estimates. These surveys could not adjust for such bias in the way that we did as they had only limited information on non-responders. There is some evidence that participation in surveys with an explicit focus on sexual health or chlamydia testing is related to individual perceptions of sexual risk [5], [23], [24]. This may lead such surveys to overestimate prevalence of chlamydia compared to estimates from studies with a general health focus such as ALSPAC. This consideration may be balanced by others such as the finding in the present study that some individuals at higher risk of infection were less likely to engage in testing. Similarly, in ClaSS, risk of infection was higher amongst women who were harder to engage in screening [5]. In Natsal 3, whilst nearly two thirds of young women reported testing for chlamydia in the previous year, this proportion was approximately halved amongst those with a positive test [6].

Our study provides the strongest evidence to date of social inequalities in the risk of chlamydia [7]. Associations with risk of infection were observed for household-level measures of socioeconomic position collected in early life and educational and neighbourhood deprivation measures taken in early and late teens. Previous studies using income, employment or area-based measures and associations with chlamydia infection have reported inconclusive results. Across different studies, chlamydia has been found to be associated with neighbourhood deprivation [6] and unemployment [25] and not associated with inability to pay utilities [25], income [26], social class [4] or neighbourhood deprivation [27] We found more consistent associations between chlamydia infection and income, social class and neighbourhood deprivation. In our study, young person's educational attainment and their mother's qualifications were strongly associated with chlamydia infection. Associations with educational level reached were found for both men and women in the Netherlands [28], women but not men in France [29], and men but not women in the USA [25]. After adjusting for reported number of sexual partners in our study, associations with maternal education and young person's education remained strong or increased, suggesting that health inequalities in chlamydia infection are not primarily explained by social variations in sexual behaviour. Similar findings have been made when adjusting educational measures for sexual behaviour in some other studies [25], [29].

This study provides an important contribution to knowledge about prevalence and social patterning of chlamydia infections among young people in England. It provides clear empirical evidence of the extent to which bias may have influenced prevalence estimates from other recent studies. Given these concerns around bias we think that evidence of lower population prevalence from our study should be interpreted cautiously. Nevertheless such evidence is in keeping with other recent evidence from the UK and may plausibly reflect an impact of UK chlamydia control programmes [21], [22]. Whilst this may be taken as encouraging by policy makers, our evidence of social inequalities in both participation in chlamydia testing and in prevalence of chlamydia should sound a note of caution. All effective health interventions have the capacity to increase health inequality because of the long recognised “inverse care” principle [9]. Reducing health inequalities, integrating screening into wider sexual health service provision and increasing screening in primary care are priorities in the new National Framework for Sexual Health [30]. There were cases of chlamydia infection at all levels of socioeconomic position in our study, indicating that reducing chlamydia prevalence and inequalities in the burden of infection requires proportionate universalism [31], ensuring that chlamydia testing services are available to all young people but intensifying efforts to promote coverage and uptake among those who are disadvantaged. The NCSP is taking efforts to achieve this, with greater provision of screening services in disadvantaged areas early in the programme's implementation [32], [33]. However, recent evidence shows uptake of screening services is similar across all levels of neighbourhood deprivation [6] despite the social gradient in infections, demonstrating that there may be a need for greater efforts to increase uptake among disadvantaged young people. General practice may have an important role to play given its high accessibility even in disadvantaged communities and there is some evidence that case finding in primary care can be increased through behavioural interventions aimed at primary care teams [34].
